# Relationships of Stresses on Alveolar Bone and Abutment of Dental Implant from Various Bite Forces by Three-Dimensional Finite Element Analysis

**DOI:** 10.1155/2020/7539628

**Published:** 2020-02-19

**Authors:** Xiaoning Kang, Yiming Li, Yixi Wang, Yao Zhang, Dongsheng Yu, Yun Peng

**Affiliations:** ^1^Guanghua School of Stomatology, Hospital of Stomatology, Sun Yat-Sen University, Guangdong Provincial Key Laboratory of Stomatology, Guangzhou, China; ^2^Guangdong Province Key Laboratory of Brain Function and Disease, Zhongshan School of Medicine, Sun Yat-Sen University, China; ^3^Zhongshan School of Medicine, Sun Yat-sen University, Guangzhou, China

## Abstract

Occlusal trauma caused by improper bite forces owing to the lack of periodontal membrane may lead to bone resorption, which is still a problem for the success of dental implant. In our study, to avoid occlusal trauma, we put forward a hypothesis that a microelectromechanical system (MEMS) pressure sensor is settled on an implant abutment to track stress on the abutment and predict the stress on alveolar bone for controlling bite forces in real time. Loading forces of different magnitudes (0 N–100 N) and angles (0–90°) were applied to the crown of the dental implant of the left central incisor in a maxillary model. The stress distribution on the abutment and alveolar bone were analyzed using a three-dimensional finite element analysis (3D FEA). Then, the quantitative relation between them was derived using Origin 2017 software. The results show that the relation between the loading forces and the stresses on the alveolar bone and abutment could be described as 3D surface equations associated with the sine function. The appropriate range of stress on the implant abutment is 1.5 MPa–8.66 MPa, and the acceptable loading force range on the dental implant of the left maxillary central incisor is approximately 6 N–86 N. These results could be used as a reference for the layout of MEMS pressure sensors to maintain alveolar bone dynamic remodeling balance.

## 1. Introduction

Dental implants have been commendably and effectively used in recent years to treat missing teeth owing to their long-term clinical success rate [1–3]. Although the success rate of dental implant therapy is high, the healing mechanism between the alveolar bone and the titanium implant fixture was replaced with osseointegration [4]. There are some vulnerabilities due to the lack of the periodontal ligament (PDL) for osseointegration [5, 6]. Therefore, the perception ability of the dental implant is deficient which increases the risks of bone resorption or degeneration due to inappropriate bite forces [7].

The biting force on the crown is transferred to the alveolar bone surrounding the dental implant, producing different stimuli to the alveolar bone. The mechanical stress has positive and negative impacts on alveolar bone remodeling [8–12]. Typically, it is considered to be favorable to alveolar bone remodeling when the strain is in the range of approximately 50–1500 microstrain [10]. The strain on the bone was considered overloading at 1500–3000 microstrain [13, 14], which could cause microdamage in the bone. When the repeated stress exceeded 3000 microstrain on the bone, deformations and middle-damage could occur, leading to fatigue failure of dental implant treatment [13]. In addition, bone fracture occurs suddenly with a force greater than 25,000 microstrain. Meanwhile, Rieger et al. also reported that functional stresses on the cortical bone ranging from 200 psi (1.37 MPa) to 700 psi (4.83 MPa) were able to maintain the existing alveolar bone height of dental implants [15, 16].

Recently, scientists have explored various methods to monitor bite forces *in vivo* and *in vitro*. These strategies can be divided into two categories. One is based on bionics, which focuses on reconstructing a biological structure similar to the periodontal membrane between the titanium implant and alveolar bone [4, 17, 18]. To date, embryonic dental follicle tissue [6] and various cell sheets [17, 19] have been used to regenerate a living periodontium around the implant. However, the sensitivity of these methods is not satisfactory, and they have high risks of carcinogenicity [20, 21]. The other strategy is to measure the bite force using instruments, such as a gnathodynamometer [22], bite forks [23], foil transducers [24], strain gauge transducers [25], force sensing resistors [26], hydraulic occlusal force gauges [27], optical fiber sensors [28, 29], and other computational methods [30]. However, these instruments are limited to a small number of discrete and static jaw positions, and it is difficult to ensure accuracy and repetition in real time. Therefore, new instruments that monitor bite forces and prevent bone resorption are required.

Recently, with the development of new technology and semiconductor materials, there have been breakthroughs for the MEMS pressure sensor. It is widely used in numerous fields because of its small size and other characteristics [31–33]. For medical applications, MEMS pressure sensors are increasingly used to control and monitor the pressure, intraocular pressure, etc., of the body [34–37], and the clinical effect evaluation is good.

In this study, we proposed a hypothesis that MEMS pressure sensors settled on the abutment were used to monitor the bite force in real time and alert patients when the biting force exceeded the appropriate range. To find the appropriate range of biting force suitable for alveolar bone remodeling, we should determine the quantitative relation between the loading force and stress on the abutment and alveolar bone. Specifically, by 3D FEA and Origin 2017 software, the quantitative relation between the loading forces and stress on the abutment and alveolar bone was evaluated, and the appropriate loading force range that benefits alveolar bone remodeling was found to be 6 N–86 N. Our work could be used as a reference for the layout of the MEMS pressure sensor in clinical treatment.

## 2. Materials and Methods

### 2.1. Patient Condition and CAD Modeling

**Figure 1:**
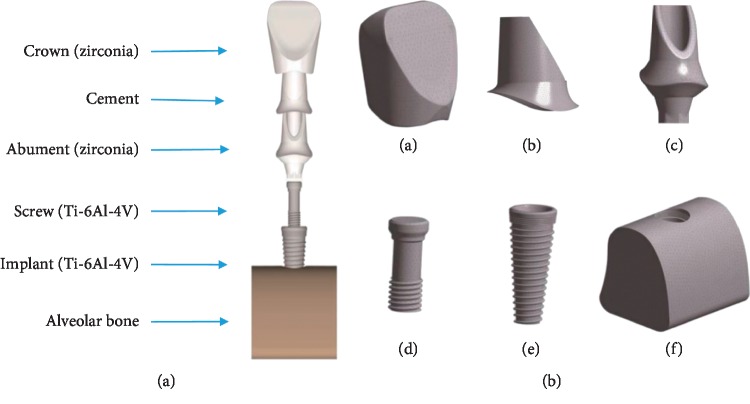
(a) 3D model of the implant system. (b) Finite element models of crown (a), cement (b), abutment (c), screw (d), implant (e), and alveolar bone (f).

A patient with a dental implant on the left maxillary central incisor was chosen. A NobelReplace Conical Connection system *φ*4.3 × 13 mm implant (Nobel Biocare®, Switzerland) was embedded in the maxillary, and a straight zirconia esthetic abutment (Nobel Biocare®, Switzerland) and zirconia crown were placed on the dental implant. The minimum alveolar thicknesses on the buccal and palatal side of the implant were 2.1 mm and 3.3 mm, respectively. The cement thickness layer between the abutment and crown was assumed to be 60 *μ*m to simulate clinical conditions [38]. The porcelain thickness of the crown and abutment was 1.5–0.5 mm [38]. The alveolar bone around the implant included a spongy center and a 1.5 mm thickness of cortical bone on the exterior. First, we imported the cone-beam (CB) CT scan images of the patient with a format of DICOM to Mimics 16.0 software sequentially. Second, we set the view orientation and defined the sagittal and coronal planes and cross section. We obtained grayscale images of the implant, crown, and alveolar bone at the interface. To improve the resolution and smoothness, we preprocessed the images. Based on the grayscale values of various parts on the image, the thresholding command was used to set the matching grayscale range to obtain the counterparts in modeling. Third, the self-extraction and filling functions and erasure were applied to ameliorate the image quality layer by layer. Finally, the rough model was exported and saved as an STL file. To further repair the rough model, we imported the STL file to 3-MATIC software. Triangular surface subdivision, noise reduction, smoothing, and accurate surface processing were conducted. Subsequently, the corresponding 3D solid models were, respectively, exported (Supplementary Materials ([Supplementary-material supplementary-material-1])) and assembled in Pro/E5.0 software, and IGES format files were exported (Figure 1(a)).

### 2.2. Finite Element Analysis

#### 2.2.1. Finite Element Modeling

The 3D model was transferred to ANSYS software and was divided into element meshes [30, 39, 40]. The 3D FEA model consisted of a total of 344,446 four-node tetrahedron elements: 54,132 elements for the implant, 42,945 elements for the abutment, 12,298 elements for the abutment screw, 80,212 elements for the crown, 31,028 elements for the cement, and 123,831 elements for the bone. Different parts of the finite element model are shown in Figure 1(b). The implant had 100% osseointegration with the alveolar bone, and the abutment was fixed to the implant with a torque of 35  Ncm [38, 39]. All models were considered homogeneous, isotropic, and linear elastic. The Young modulus and Poisson ratio of each material used in this study are listed in Table 1 [38, 41–43].

#### 2.2.2. Loading Conditions on the Crown

**Figure 2:**
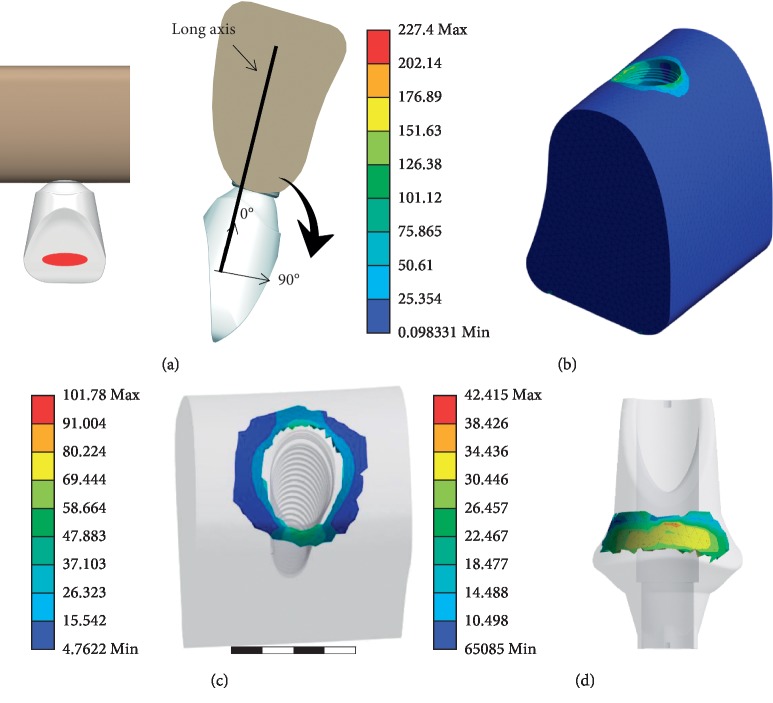
The condition of loading force and selection of the region of interest (ROI) of the alveolar bone and abutment. (a) Loading force position and loading force angle. (b) The region of stress concentration on the alveolar bone. (c) The ROI of the alveolar bone. (d) The ROI of the abutment.

The acting point of the loading force was placed on the lingual side 1/3 closer to the biting surface and the center of the mesial and distal sides (Figure 2(a)). The loading angles ranged from 0° to 90° with an interval of 5° relative to the occlusal plane. The values of the loading forces were 1 N to 100 N with an interval of 1 N.

#### 2.2.3. Selection of the Region of Interest (ROI) of the Alveolar Bone and Abutment

The region surrounding the implant is the most stressed and vulnerable. Thus, this area was chosen as the ROI in our model. In addition, in our modeling, the maximum stress was mainly distributed on the cortical bone (Figure 2(b)), which is consistent with previous studies [42, 44–46]. Therefore, the stress on the cortical bone of the ROI (Figure 2(c)) was analyzed. The upper slope of the abutment was smooth and flat, and it was considered suitable for placement the MEMS pressure sensor, and the ROI on the abutment was examined (Figure 2(d)). The stress distribution on the ROI of the cortical bone and abutment with different loading forces was evaluated using a Von Mises analysis. The emulated data in these areas were analyzed.

## 3. Results

### 3.1. Quantitative Relation between the Loading Force and Stress on the Cortical Bone and Abutment

**Figure 3:**
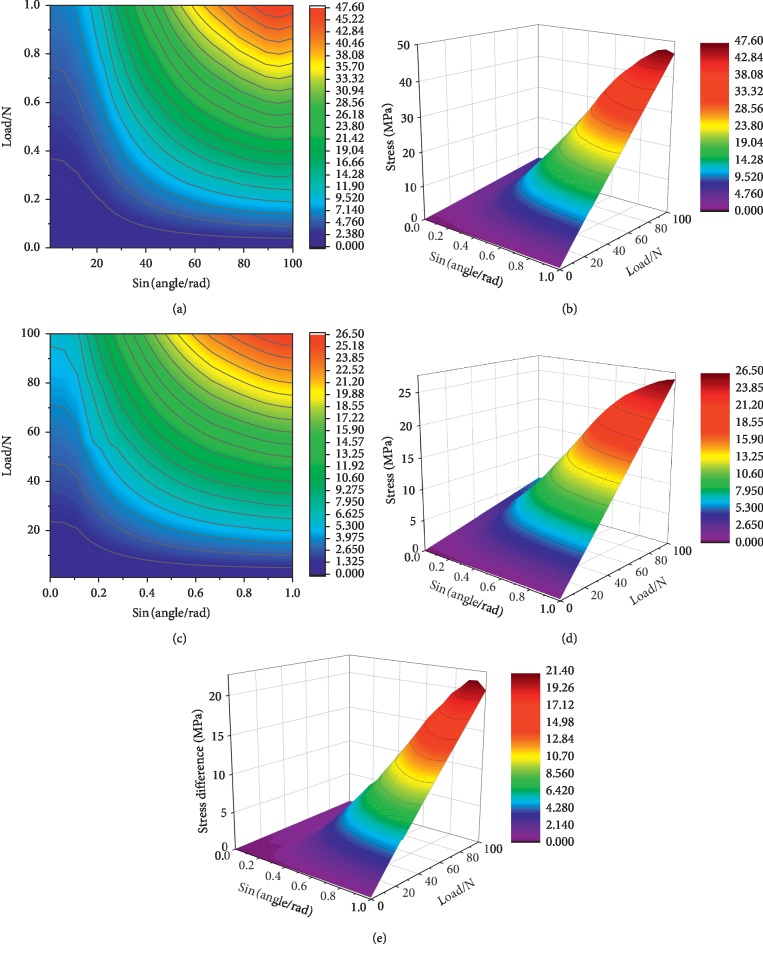
Quantitative relation between the loading force and stress on the cortical bone and abutment. (a) Von Mises contour map of the ROI of the abutment under different magnitudes and angles of loading forces. (b) 3D surface of the abutment under different magnitudes and angles of loading forces. (c) Von Mises contour map of the ROI of the alveolar bone under different magnitudes and angles of the loading forces. (d) 3D surface of the alveolar bone under different magnitudes and angles of loading forces. (e) 3D surface of the stress differences of the abutment and alveolar bone under different magnitudes and angles of loading forces.

The quantitative relation between the loading force and stress on the ROI of the cortical bone is depicted in a 3D curved surface (Figure 3(a)) and contour (Figure 3(b)). The quantitative relation between the loading force and stress on the ROI of the abutment is shown in a 3D curved surface (Figure 3(c)) and contour (Figure 3(d)), respectively. The quantitative relation between the loading force and numerical difference of the stress on the abutment and cortical bone is shown in a 3D curved surface (Figure 3(e)).

The emulated results showed that the stress on the cortical bone or the abutment increased with the angles of the loading forces when the magnitude of the loading force was fixed. In addition, when the angle of the loading force was invariable, the stress on the cortical bone and abutment increased with increasing loading forces. Similarly, the numerical difference of stress on the abutment and cortical bone increased with the angles and magnitude of the loading force.

### 3.2. 3D Curved Surface Equation Derivation and Accuracy Prediction

To further evaluate the quantitative relation between the loading force and stresses on the cortical bone and abutment, Origin 2017 software was used to process the data and derive equations between them. The accuracy of the equations was predicted.

#### 3.2.1. Loading Force and Stress on the Cortical Bone

Origin 2017 software was utilized to analyze the emulated data. The Poly2D model fitted well with the data. The 3D surface equation between the loading forces and stress on the cortical bone is shown as

(1) $$\begin{array}{c} z=2.28312+0.82124x-0.0184\;\sin\;y-3.94944x^{2}+4.43792\times 10^{-4}{\left(\sin\;y\right)}^{2}+0.28129x\;\sin\;y. \end{array}$$

Here, *X* is the magnitude of the loading force (0–100 N), *Y* is the loading force angle (0–90°), and *Z* is the Von Mises stress on the cortical bone (MPa).

Based on the above equation, the values of the stress on the cortical bone could be calculated from the loading force. Thereafter, two methods were used to verify the correlation and prediction accuracy of this model. First, Origin 2017 software was used to conduct linear fitting between the calculated value and emulated data of the Von Mises stresses on the cortical bone. The obtained equation is

(2) $$\begin{array}{c} y=2.10498+1.12649x, \end{array}$$

where *Y* is the calculated Von Mises stress value (MPa) and *X* is the simulated Von Mises stresses value (MPa).

This equation shows a high relevance between the calculated value and emulated data.

Second, the prediction accuracy was determined by the correlation coefficient (*R*) and average absolute relative error (AARE) [47]. The parameter *R* provided information on the strength of the linear relation between the experimental and predicted values. AARE was calculated by a term comparison of relative errors and could be used to measure the unbiased statistical parameters of the predictability of the equation. They are, respectively, expressed as

(3) $$\begin{array}{c} R=\frac{\sum_{i=1}^{N}\left(X_{i}-\bar{X}\right)\left(Y_{i}-\bar{Y}\right)}{\sqrt{\sum_{i=1}^{N}{\left(X_{i}-\bar{X}\right)}^{2}}\sqrt{\sum_{i=1}^{N}{\left(Y_{i}-\bar{Y}\right)}^{2}}},\\ \text{AARE}=\frac{1}{N}\sum_{i=1}^{N}\left|\frac{Y_{i}-X_{i}}{X_{i}}\right|\times 100\%, \end{array}$$

where *X*_*i*_ and *Y*_*i*_ are the emulated and predicted stresses, respectively; $\bar{X}$ and $\bar{Y}$ are the average of *X*_*i*_ and *Y*_*i*_, respectively, and *N* is equal to the number of emulated data.

The results show that the value of *R* is 0.990. Thus, there is a close correlation between the emulated data and calculated value. Here, AARE is 0.27; therefore, the equation provides a sound prediction of the stress on the cortical bone caused form different loading forces.

#### 3.2.2. Loading Force and Stress on the Abutment

To analyze the emulated data between the loading force and stress on the abutment, Origin 2017 software was utilized, and the equation is shown as follows:

(4) $$\begin{array}{c} z=2.47356+0.02119x-4.51317\;\sin\;y-5.29612\times 10^{-4}x^{2}+4.99362{\left(\sin\;y\right)}^{2}+0.53559x\;\sin\;y. \end{array}$$

Here, *X* is the magnitude of the loading force (0–100 N), Y is the loading force angle (0–90°), and *Z* is the Von Mises stress on the abutment (MPa).

The calculated value and emulated data of the Von Mises stress on the abutment was fitted linearly, and the relation was obtained as follows:

(5) $$\begin{array}{c} y=0.5998+1.09702x. \end{array}$$

Here, *Y* is the calculated Von Mises stress value (MPa) and *X* is the simulated Von Mises stress value (MPa).

Here, *R* was 0.994, representing a good correlation between the calculated value and emulated data. Meanwhile, AARE was only 0.375, indicating that the equation could adequately predict the stress on the abutment from various loading forces.

#### 3.2.3. Quantitative Relation of the Loading Force Differences on the Abutment and Cortical Bone

To analyze the emulated data for the numerical loading force differences on the abutment and cortical bone, Origin 2017 software was utilized, and the 3D surface equation is shown as follows:

(6) $$\begin{array}{c} z=0.28756+0.9492x-0.02659\;\sin\;y-1.47644x^{2}+9.91986\times 10^{-4}{\left(\sin\;y\right)}^{2}+0.25459x\;\sin\;y. \end{array}$$

Here, *X* is the loading force (0–100 N), Y is the loading force angle (0–90°), and *Z* is the difference of the Von Mises stress on the abutment and cortical bone (MPa).

The calculated value and emulated data of the differences were fitted linearly, and the obtained equation is as follows:

(7) $$\begin{array}{c} y=1.359+1.20x. \end{array}$$

Here, *Y* is the calculated Von Mises stress value (MPa) and *X* is the emulated Von Mises stress value (MPa).

Here, *R* is 0.95304, and AARE is 0.234. The results were similar to those described above. There was a good correlation between the loading force difference on the abutment and cortical bone.

#### 3.2.4. Appropriate Loading Force for Alveolar Bone Remodeling

Utilizing our 3D surface equation, we can calculate the stress on the alveolar bone and abutment on the dental implant of the central incisor from the loading forces, combined with the documented data, which is appropriate for alveolar bone remodeling [15, 48, 49]. The appropriate range of stress on the abutment suitable for alveolar bone remodeling is 1.5 MPa–8.66 MPa, and the optimum occlusal force range of the dental implant of the left maxillary central incisor was approximately 6 N–86 N (Table 2).

## 4. Discussion

The dental implant is liable to alveolar bone resorption caused by improper biting forces owing to a lack of the periodontal membrane [4]. Numerous bite force measurements are conducted by various instruments [30, 46, 50]. However, these instruments are limited because it is difficult to ensure the shortage of accuracy and repetition in real time [30, 46, 51]. Based on our hypothesis, MEMS pressure sensors were placed on the abutment to monitor the bite force in real time and compensate for the above latent shortcomings to a certain extent. This study aimed to determine the relation between the loading force and stress on the abutment and alveolar bone, providing references for layout of the MEMS pressure sensor.

Various methods are used to analyze the biomechanical responses of dental structures. FEA is widely used in dental biomechanics for the noninvasive assessment of the bite force, strains, stress, and displacement in the dental structures [52–55]. Thus, in this study, we used FEA to evaluate the concentration and distribution of stress on the implant and adjacent alveolar bone tissue. The relation of the stress on the alveolar bone and abutment from various loading forces was derived using Origin 2017.

Our emulated results demonstrated that the maximum stress on the alveolar bone was on the cortical bone region around the implant, which is consistent with other studies [42, 44–46].

Next, we obtained the relation between the loading force and stress on the alveolar bone and abutment with sine function. Based on Figures 3(a) and 3(b), the stress values of the alveolar bone or abutment gradually increased with the increase in the loading force. When the magnitudes of the loading force were immobile, the angle and the corresponding stress values were larger [56, 57]. The reason is that a larger torque could be generated by a force at a larger angle [42, 58, 59].

In addition, based on the fitting equation and correlation coefficient (*R*) and average absolute relative error (AARE), the prediction accuracy was further confirmed. According to our 3D surface equation, we calculated the appropriate range of the loading force set on the abutment which is 6 N–86 N for anterior dental implant. If the biting force is less than this range, it will cause degeneration of the alveolar bone. When it exceeds this range, the alveolar bone will be damaged. Therefore, this result provides a reference for eliminating occlusal interferences and establishing an optimum occlusal relation for durability.

In this study, we proposed a hypothetical implant bite force-detection system, a MEMS pressure sensor, to improve the success rate of the traditional dental implant. The precision, repeatability, and comfort of this instrument are expected to be greatly improved. Additionally, it was placed on the abutment to prevent the possibility of bio-incompatibility between the sensor and oral cavity by avoiding contact with the external oral cavity. It was encompassed completely by the crown to improve corrosion resistance. Finally, if performance problems were to occur, it could be repaired or replaced.

The loading force affects the success of the implant and plays a critical role in other oral-related diseases, including brain degeneration diseases such as Parkinson's and Alzheimer's diseases [60, 61]. The real-time monitoring data of the bite forces can be used to evaluate dentures, dentition, temporomandibular joint function, the results of orthognathic surgery, the functional performance of prosthetic devices, and the chewing law of bruxism.

However, developing an accurate loading force monitoring model is complicated. It is affected by numerous factors. First, the dynamics of loading on the alveolar bone, including the strain rate, loading frequency, loading time, and bone mineral density, which determine the mechanical parameters in osteogenic loading and the remodeling stages, should be considered [38, 62, 63]. Second, the bite force varies with different devices, including the simulation mesh division, angle and force degree interval selection, and potential formulas and algorithms [46], which could add error in our system. The detection system should be customized and corrected according to different conditions. Third, in clinical conditions, the osseointegration levels at the peri-implant area, the timing and functional distribution of the force, and the nonhomogeneous, anisotropic, and nonlinear responses of the alveolar bone should be considered. Finally, animal and clinical experiments should be conducted to verify the emulated data in future work.

## 5. Conclusion

In this study, the relations between the loading force and stress on the abutment and alveolar bone were evaluated for our hypothesis. The appropriate range of stress on the abutment suitable for alveolar bone remodeling is 1.5 MPa–8.66 MPa, and the appropriate range of the loading force is 6 N–86 N, which could provide a basis for future layout of the MEMS pressure sensor in clinical treatment.

## Figures and Tables

**Table 1:** Mechanical properties of materials.

	Crown and abutment (zirconia)	Implant and screw (Ti-6Al-4V)	Cement	Cancellous bone	Cortical bone
Young's modules (Pa)	2.1 × 10^11^ [41]	1.1 × 10^11^ [38]	1.4 × 10^10^ [38]	1.37 × 10^9^ [42, 43]	1.37 × 10^10^ [43]
Poisson's ratio	0.3 [41]	0.32 [38]	0.35 [38]	0.3 [43]	0.3 [43]

**Table 2:** The predicted range of stresses on abutment and alveolar bone from their corresponding magnitudes and directions of loading forces.

Angle (degree)	Loading forces (N)	Stress (Mpa)	
		Abutment	Alveolar bone
0	25–86	1.58–5.45	1.40–4.80
5	25–85	1.63–5.55	1.42–4.82
10	22–76	1.66–5.72	1.39–4.80
15	17–57	1.50–5.73	1.43–4.80
20	14–46	1.74–5.73	1.47–4.82
25	11–38	1.81–6.25	1.37–4.74
30	10–33	2.03–6.71	1.43–4.73
35	9–30	2.17–7.22	1.45–4.83
40	8–27	2.21–7.46	1.42–4.80
45	8–25	2.48–7.74	1.54–4.82
50	7–23	2.39–7.84	1.45–4.75
55	7–22	2.59–8.12	1.53–4.81
60	6–21	2.37–8.30	1.37–4.81
65	6–21	2.51–8.36	1.43–4.76
70	6–19	2.62–8.31	1.47–4.85
75	6–19	2.72–8.62	1.51–4.79
80	6–18	2.80–8.39	1.55–4.65
85	6–18	2.85–8.56	1.58–4.73
90	6–18	2.89–8.66	1.59–4.77

## Data Availability

The data used to support the findings of this study are included within the article.

## Supplementary Materials

Supplementary Materials (1) The 3D structure of implant parts: the 3D models of the implant, screw, abutment, cement, crown, and alveolar bone and their connection are shown. Crown: a 3D modeling of the dental zirconia implant crown on the left maxillary central incisor was simulated and built, and the porcelain thickness of the crown and abutment was 1.5–0.5 mm. Abutment: a straight zirconia esthetic abutment (Nobel Biocare®, Switzerland) was simulated and built. Cement: the cement thickness layer between the abutment and crown was assumed to be 60 *μ*m to simulate clinical conditions. Implant: a NobelReplace Conical Connection system *φ*4.3 × 13 mm implant (Nobel Biocare®, Switzerland) was simulated and built. Screw: screws are used to secure the abutment and implant. Alveolar bone: the minimum alveolar thicknesses on the buccal and palatal side of the implant were 2.1 mm and 3.3 mm, respectively. The alveolar bone around the implant included a spongy center and a 1.5 mm thickness of cortical bone on the exterior. (2) Processing of 3D images: first, we imported the cone-beam (CB) CT scan images of the patient with a format of DICOM to Mimics 16.0 software sequentially. Second, we set the view orientation and defined the sagittal and coronal planes and cross section. We obtained grayscale images of the implant, crown, and alveolar bone at the interface. To improve the resolution and smoothness, we preprocessed the images. Based on the grayscale values of various parts on the image, the thresholding command was used to set the matching grayscale range to obtain the counterparts in the modeling. Third, the self-extraction and filling functions and erasure were applied to ameliorate the image quality layer by layer. Finally, the rough model was exported and saved as an STL file. To further repair the rough model, we imported the STL file to 3-MATIC software. Triangular surface subdivision, noise reduction, smoothing, and accurate surface processing were conducted. Subsequently, the SLDPRT files of the corresponding 3D solid models were, respectively, exported.
